# Referrals and Black-White Coronary Heart Disease Treatment Disparities: A Qualitative Study of Primary Care Physician Perspectives

**DOI:** 10.1007/s11606-024-09175-x

**Published:** 2024-11-13

**Authors:** Nabeel Qureshi, Sandra Berry, Cheryl L. Damberg, Ben Gibson, Ioana Popescu

**Affiliations:** 1 RAND Corporation, Los Angeles, USA; 2 David Geffen School of Medicine, UCLA, Los Angeles, USA

**Keywords:** primary care, cardiology, physician referral, hospital quality, coronary heart disease, disparities

## Abstract

**Background:**

Black-White coronary heart disease (CHD) treatment disparities are well documented, especially regarding the use of high-quality hospitals. Physician referral networks may play a role.

**Objective:**

To understand how primary care physicians (PCPs) make specialty referrals for CHD treatment and how referrals may contribute to treatment disparities.

**Design:**

Qualitative study using semi-structured interviews and focus group discussions.

**Participants:**

We purposively recruited 45 PCPs (50 invited, 90% response rate) in three metro areas with high Black-White segregation of cardiac care networks (New York City; Chicago; Atlanta).

**Approach:**

We developed the focus group discussion guide from interviews and current literature. We conducted two focus groups per metro area via Zoom. Two expert team members independently coded the transcripts using inductive techniques and analyzed focus group content and themes using Dedoose.

**Key Results:**

Most participants were male (62.2%), White (57.8%), and practiced for at least 23 years. We identified several recurrent themes for factors influencing cardiology referrals. The most frequently mentioned themes were heavy reliance on professional networks, specialist availability, timeliness, communication style, patient geographic and economic constraints, and patient preferences. PCPs used anecdotal and not data-driven evidence to assess hospital quality and viewed Black-White differences in high-quality hospital use as due to patient economic status and preferences or differences in hospital access and provider referral bias.

**Conclusion:**

PCPs’ referral decisions for CHD treatment are primarily driven by access to specific professional networks and the socioeconomic circumstances of their patients. Nevertheless, PCPs strive to make the best available decisions, leaning into their networks and honoring patient preferences. While PCPs acknowledged existing disparities, they attributed them to patient and system factors rather than provider referral bias. Mitigating disparities will require interventions to improve minority-serving providers’ formal and informal connections with high-quality specialists and hospitals, address patient socioeconomic constraints, and train providers to recognize their potential biases and misconceptions.

**Supplementary Information:**

The online version contains supplementary material available at 10.1007/s11606-024-09175-x.

## INTRODUCTION

Despite sustained policy efforts, Black-White disparities in coronary heart disease (CHD) treatment remain substantial, especially for advanced, elective procedures such as coronary artery bypass (CABG) surgery.^1–5^ A growing body of evidence shows that disparities may be due in part to differences in the quality of hospitals where Black and White patients receive treatment.^6–12^ Differences persist even after accounting for geographic proximity,^13–16^ suggesting that non-geographic factors play an important role. In particular, Black and White patients may use different physician referral networks, as demonstrated by recent work showing that processes of care, including specialty referrals for Black and White patients, are segregated across hospitals,^17^ provider teams,^18^ and within physicians.^10,19,20^ While these studies may shed critical light on observed referral patterns, they stop short of examining the underlying physician decision-making processes. A better understanding of how physicians make referrals is key to identifying barriers to high-quality care.

The reasons underlying physicians’ referral decisions have been previously published. Early work by Shortell et al. demonstrated that physicians make referrals weighing potential benefits (e.g., quality of care) and costs (e.g., loss of reputation) based on prior knowledge and personal experience.^21,22^ Decades later, this model largely holds true,^23–27^ with several studies finding that informal relationships between physicians,^27^ anecdotal evidence on the quality of care provided by specialists,^25,26^ and patient convenience^24,27^ are still the main drivers of referral decisions. Less is known about factors specific to hospital referral but extant evidence suggests that similarly subjective factors (e.g., familiarity with the hospital, admitting arrangements and patient preferences) play key roles, whereas published performance measures are generally not considered.^28^

The contribution of physician referral decisions to disparities in access to cardiologist and hospital services for CHD patients has been insufficiently explored. Prior research has documented implicit bias in physician decision-making for an array of services including referral for coronary procedures but its relationship to outcome disparities has been inconsistent.^29^ A complete picture of how physician referral decisions contribute to disparities in CHD treatment has yet to emerge.

The current study begins to address this knowledge gap by focusing on the PCP to cardiologist referral, the most common entry point into the referral system for CHD treatment.^30^ As such, the cardiology referral may be a key first step to influence access to high-quality specialists and hospitals for advanced diagnostic tests and procedures. Specifically, we sought to understand the factors that inform PCPs’ choices of cardiologists, and how referrals may lead to disparities in the quality of hospitals used by their patients. Because health system integration often provides streamlined referral pathways, we were primarily interested in highlighting the referral considerations of physicians who practiced independent of large systems. We accomplished our objective through focus groups that encompassed an initial discussion of factors driving PCPs’ referral to cardiologists followed by a case study using the specific example of CABG, a common, referral-dependent cardiac procedure with well-documented hospital-based disparities.^31–33^

## METHODS

We conducted three semi-structured pilot interviews and six focus group discussions with PCPs in three US health care markets (New York City, NY; Chicago, IL; Atlanta, GA) to elicit their opinions on referral processes. We selected these markets due to large minority populations and high Black-White physician network segregation for cardiac care. Details of the segregation metrics we used have been published elsewhere.^34^ A brief summary of segregation levels in study markets is provided in Fig. S1 and Table S1 (Online Supplement). We recruited two focus groups per market, each consisting of only physicians practicing in that market.

### Participant Selection

We employed a private vendor using a proprietary database to identify and recruit study PCPs. The database is supplied by multiple private and public data sources and contains information on physicians’ specialty, practice location and type (e.g., independent group vs. integrated health system), years in practice, and sociodemographic characteristics. PCPs were eligible for the study if they had at least 3 years of practice (to ensure established referral patterns); were not formally affiliated with large integrated health systems; treated at least 25% Black patients during the prior 12 months; and recalled at least one patient who had elective CABG at a high-quality hospital during the previous year. High quality was ascertained as hospital mortality in the lowest quintile, using the public Medicare CABG hospital mortality measure. Physicians were provided with a list of these hospitals in their market area.

Overall, the vendor identified a sample of 72 PCPs who met the inclusion criteria, of which 50 were recruited via email. A total of 45 PCPs participated across focus groups (between six and eight in each focus group); five eligible participants were no-show.

### Study Design

Development of the discussion guide (see Table S2, Online Supplement) was informed by prior literature^21,24,27,28^ and by three semi-structured pilot interviews conducted with PCPs in the study areas. Each focus group lasted 90 min and was conducted online via Zoom and audio recorded and transcribed with participant consent. Discussions were led by researchers with prior experience conducting physician focus group, quality of care, and disparities research. Each focus group included two facilitators (SB, female, expert in qualitative research and focus group facilitation; IP, female, PCP and expert in health care disparities research) to ensure that we elicited balanced perspectives. Overall, discussions centered on non-emergent CHD, for which referrals are likely to play a central role. Focus group sessions started with a 1-h discussion of general factors driving referral decisions for CHD treatment. This was followed by a 30-min discussion in which participants were presented with the team’s data showing disparities in the quality of hospitals where Black and White Medicare patients receive CABG surgery (Fig. S2, Online Supplement). This discussion aimed to elicit participant PCPs’ opinions on reasons why disparities occur, including the role of physician referrals.

Participants were compensated $400 for their time and participation. This work was approved by the RAND Human Subjects Protection Committee.

### Data Analysis

We used inductive techniques to conduct content and thematic coding and analysis of focus group discussions following established methods.^35,36^ Two coders (IP and NQ) reviewed the transcripts and applied preliminary codes to each transcript, adding new codes to a preliminary codebook based on emerging themes and insights. The coding team met regularly to review progress, update the codebook, conduct targeted coding and recoding, and address inconsistencies, thus ensuring consistent coding practices. After finalizing the codebook, we performed an inter-rater reliability test using one completely coded transcript. The calculated kappa score was 0.72, indicating good agreement among coders.^37^

We reviewed coded excerpts holistically and by focus group to identify themes and subthemes. Quotations are used to illustrate themes and subthemes. Analysis was conducted in Dedoose.^38^ All data is reported in line with the Consolidated Criteria for Reporting Qualitative Research (COREQ) and included in Table S3 (Online Supplement).^39^

## RESULTS

### Sample Characteristics

The characteristics of participating PCPs are described in Table 1. Most participants were male (62.2%), White (57.8%), and practiced for at least 23 years. Participant specialty was either internal medicine (60%) or family medicine (40%).

**Table 1:** Demographic Characteristics of Primary Care Physicians Participating in Focus Groups

Demographics	Atlanta, GA	New York City, NY	Chicago, IL
Count	15	16	14
Gender			
Male	46.7% (7)	81.3% (13)	57.1% (8)
Female	53.3% (8)	18.8% (3)	42.9% (6)
Race			
White	33.3% (5)	62.5% (10)	78.6% (11)
Black	53.3% (8)	12.5% (2)	0% (0)
Asian	6.7% (1)	12.5% (2)	14.3% (2)
Other	6.7% (1)	12.5% (2)	7.1% (1)
Specialty			
Internal medicine	26.7% (4)	93.8% (15)	57.1% (8)
Family medicine	73.3% (11)	6.3% (1)	42.9% (6)
Years in practice (mean)*	23.0 (7.9)	23.6 (6.8)	23.4 (9.4)
% Medicare patients (mean)*	37%	37%	56%
*N* CABG patients during past year	15.5 (13.1)	35.3 (45.1)	29.3 (24.4)

^*^Self-reported responses

### General Referral Discussion Themes

We identified several recurrent and unifying themes surrounding drivers of cardiology referrals. A summary of these themes is provided in Table 2; a complete list of quotes is provided in Table S4 (Online Supplement). For each quote, we provide PCP gender, unique identifier, and market location (e.g., M[ale]MD[ID]54; [market] New York, NY).

**Table 2:** General Discussion Themes and Subthemes

Theme	Subthemes	Representative quotations
The importance of professional networks	PCPs deeply rely on informal professional networks when making referral decisions	“Two cardiologists that I work with […] see probably 90 percent of my patients and I think they respect my judgment and when I ask them to see someone in a pinch, they’ll do so within 24 h. I think it depends a lot on the relationship you have with the cardiologist—if they know you and have a good relationship with you, then they’re not going to turn your referral away.”—MMD54 (New York, NY)
	Strong professional relationships are based on trust and mutual respect	“If the patients are happy with the doctor, they have a good bedside manner, that makes me happy […]. Fortunately, I have the same group of doctors for a long time.”—FMD55 (New York, NY)
	Informal referral networks are becoming increasingly unstable	“I’ve been in this area for over 20 years and have some specific doctors that I refer to […] And it’s been up and down over the years. In fact, maybe a year or two ago, it got to the point where I really didn’t have a go-to person for a while”—MMD38 (Atlanta, GA)
Valued referral provider characteristics	Desirable specialist characteristics include availability, timeliness, and good communication skills	“The first couple patients you send, you ask questions when they come back. You look at the [consult] notes and you could […] call and discuss the patient with [the cardiologist]. I think that’s good […]. So, […] if somebody communicates well with you and [takes] care of the patient […] as soon as possible, […] that tells me that I’m dealing with the correct group.”—FMD66 (Atlanta, GA)
	Diminished and delayed access to specialists is a growing referral barrier	“And it’s become a problem in […] in the last couple of years. Because before, I used to be able to call one of my favorite cardiologists and they would […] accommodate the patient. But lately, it seems…I luck out occasionally […] but for the most part, it’s going to be a wait.”—FMD67 (Atlanta, GA)
Consideration of patient circumstances	Geographic proximity and SDOH are important in the referral process	“You need to follow up with the specialist after a […] procedure[…]. [Some patients] will not follow-up. They struggle financially, they cannot get there, they don’t have supportive family. So, when you look at the whole picture, it’s still better if they are closer to home because they have better chances for follow-up.”—FMD15 (Chicago, IL)
	Patient preferences matter	“If it’s something not as urgent [*…*], I may defer to what the patient wants in the sense that sometimes they’ll want to see somebody close to their house, so we’ll try to find them somebody in their area.”—MMD53 (New York, NY)
	PCPs lean into their networks to overcome SDOH limitations	“If it’s somebody who does not have insurance and can’t afford to pay a lot, again I’ll lean on those relationships. […] I know some cardiologists that will charge lower out-of-pocket costs to people who can’t afford it, so I’ll utilize those services in those cases.”—MMD53 (New York, NY)
	Quality concerns may override patient preferences	“There are some hospitals [that] handle cardiac care better than others. So, depending upon [severity], that makes an impact on where I might direct the patient over where they feel they may be the most comfortable.”—FMD35 (Atlanta, GA)
Specialist vs. hospital choice	Good cardiologists provide the same quality of care at any hospital and are best positioned to make hospital referrals	“It’s typically the cardiologist that [..] will make the phone calls. If I have a patient that says, ‘Where is the best place to have a CABG done?’ I’ll say, ‘I’m sending you to who is the best, who is very good.’ I mean, when you say the best hospital, what is it about the hospital? It’s the physician […] that really makes the difference. It’s not just the prestige of the hospital, it’s the physician who’s doing it. So, if there’s somebody at […] our hospital that’s equally capable of doing it, I will tell the patient that you’ll get the same standard of care as you would somewhere else. But if it’s something super-specialized, then the cardiologists will make the call.”—MMD13 (Chicago, IL)

#### The Importance of Professional Networks

PCPs described referral decisions as primarily determined by the strength and quality of their professional relationships, driven in turn by trust and mutual respect. In the words of one PCP, “Two cardiologists that I work with […] see probably 90 percent of my patients and I think they respect my judgment and when I ask them to see someone in a pinch, they’ll do so within 24 h. I think it depends a lot on the relationship you have with the cardiologist—if they know you and have a good relationship with you, then they’re not going to turn your referral away” (MMD54; New York, NY).

PCPs also discussed the effects of changing professional relationships on referrals. In particular, PCPs who had to rely on informal professional networks described these as increasingly unstable (Table 2).

#### Valued Referral Provider Characteristics

PCPs described several specialist characteristics they found highly valuable when making referral decisions: availability, timeliness, reporting back, and returning patients to the PCP to ensure continuity of care. One PCP summed up the ideal referral provider as a good communicator who provides good-quality timely care to their patients: “The first couple of patients you send, you ask questions when they come back. You look at the [consult] notes and […] call and discuss the patient with [the cardiologist]. I think that’s good. […] If somebody communicates well with you and [takes] care of the patient […] as soon as possible, […] that tells me that I’m dealing with the correct group” (FMD66; Atlanta, GA).

At the same time, PCPs noted increasing barriers to cardiologist accessibility and delays in consultations due to a changing practice environment: “I find that I have difficulty. And it’s become a problem in recent years. Because before, I used to be able to call one of my favorite cardiologists and they would be able to accommodate the patient. But lately, it seems…I luck out occasionally but for the most part, it’s going to be a wait” (FMD67; Atlanta, GA).

#### Consideration of Patient Circumstances

Participant PCPs considered patient circumstances when making referral decisions and were particularly aware of their geographic and socioeconomic constraints. PCPs weighed travel distance and social determinants of health (SDOH) including supplemental insurance, social support, and transportation when making specialist and hospital referrals. One PCP discussed these constraints as unquantifiable factors that ultimately drive patient outcomes: “Social demographics. I don’t have a car. I don’t have bus money. I don’t have someone to watch my children. Or I have coexisting mental illness, things […] that you can’t quantify that definitely affect outcomes” (FMD67; Atlanta, GA).

These constraints sometimes weighed heavier in referral decisions than considerations of quality, especially for procedures that require extensive follow-up. One PCP described these sometimes-difficult decisions: “You need to follow up with the specialist after a […] procedure, whether it’s a cardiac surgeon, or a cardiologist. [Some patients] will not follow-up. […] They struggle financially, they cannot get there, they don’t have supportive family. So, when you look at the whole picture, it’s still better if they are closer to home because they have better chances for follow-up” (FMD15; Chicago, IL).

Nevertheless, PCPs worked hard to overcome limitations due to patient SDOH by leaning into their professional networks, even when these efforts were an additional burden: “If it’s somebody who does not have insurance and can’t afford to pay a lot, again I’ll lean on those relationships. […] I know some cardiologists that will charge lower out-of-pocket costs to people who can’t afford it, so I’ll utilize those services in those cases” (MMD53; New York, NY).

Beyond SDOH constraints, PCPs try to honor patients’ preferences when selecting referral providers, considering patients’ prior negative experiences, desire for language concordance, or preference for services closer to home (Table 2). Even so, PCPs did sometimes override patient preferences in favor of quality: “There are some hospitals [that] handle cardiac care better than others. So, depending upon [the patient severity], that makes an impact on where I might direct the patient over where they feel they may be the most comfortable” (FMD35; Atlanta, GA).

#### Specialist vs. Hospital Choice

Overall, participant PCPs considered hospital choice secondary to specialist choice. This was supported by PCPs’ belief that cardiologists would provide the same quality of care at any hospital and were better positioned to choose hospitals for procedures: “When you say the best hospital, what is it about the hospital? It’s the physician […] that really makes the difference. It’s not just the prestige of the hospital, it’s the physician who’s doing it” (MMD13; Chicago, IL).

### Themes Emerging from the Discussion on Disparities in High-Quality Hospital Use

In the second part of discussions, PCPs were presented with data on Black-White disparities in high-quality hospital use for CABG. This discussion segment yielded themes on hospital quality assessment and barriers to high-quality hospital use for Black patients, detailed below and in Table 3. A comprehensive list of quotes is presented in Table S5 (Online Supplement).

**Table 3:** Quality Discussion Themes and Subthemes

Theme	Subthemes	Representative quotations
Assessment of hospital quality	PCPs are unaware of published hospital quality data	“I’ve been practicing for 18 years, and I don’t really know of these ratings. And I’m embarrassed to say I don’t. But even if I did know that the ratings exist—I mean, if it was in front of me, I’d probably find some interest about it, but I still have my favorite cardiologists that I don’t have a problem with and [who have] great bedside manner. And I’m just going to stick with them, you know?”—MMD65 (Atlanta, GA)
	PCPs are distrustful of published hospital quality data	“[There is] all the data that you can get, but it’s hard to judge. I mean, even looking for a doctor for myself, it’s very hard to judge […] the information out there, what you see on a website, what’s reported, if there are ways to game systems […] So, you sort of do the best you can.”—MMD53 (New York, NY)
	PCPs employ trust-based assessments of providers over published data	“You know, those formal ratings, even though you might think they’re supposedly objective, […] if [hospitals] are taking care of much more complicated patients [ …] they’re going to have […] worse outcomes, perhaps, because of the patient population they’re taking care of. So, ratings that may seem objective aren’t necessarily all objective.”—FMD11 (Chicago, IL)
Perceptions of disparities in access to high-quality hospitals	PCPs are unaware of the magnitude of disparities in high-quality hospital use, but do agree that they seem likely	“I do believe these stats. Even if you were to put everything in equal terms, same patients with equal insurance, even sent to the same hospital… the disparity is not just with CABG and cardiac issues. If you dig [into] statistics or if you search in the medical literature, it’s across the board”—MMD54 (New York, NY)
	Disparities in high-quality hospital use are driven by patient and health system factors	“If patients are in a particular community of hospitals, and they don’t necessarily know their rating scores, they’re just going to go where [access] is offered that’s near versus driving 30 miles across town to a higher quality hospital. Again, depending on socioeconomic status and ability to pay”—F-MD64 (Atlanta, GA) “The low-quality hospitals are probably more likely in the areas where more Black people live, [and patients are likely to go] someplace that is close to them, [where…] employees […] are more likely Black as well. So, all those things come into play.”—M-MD63 (Atlanta, GA) “We have Black folks living in one area and the White folks living in the other [and] we know that certain hospitals […] might have lower quality ratings, and they tend to be in minority neighborhoods”—MMD14 (Chicago, IL)
	Provider referral bias does not play a role in high-quality hospital use disparities	“I can speak for our practice. We don’t distinguish on the basis of color. Black, white, yellow, red, purple, it doesn’t—I mean, I really don’t think so. And my referrals are based on the acuteness of the situation and the severity of the situation”—MMD22 (New York, NY)

#### Assessment of Hospital Quality

PCPs were unfamiliar with published data-driven hospital quality measures and had low trust in these measures; therefore, quality ratings were rarely if ever used to make hospital referral decisions. One participant described not using data-driven measures in their referral decisions: “I’ve been practicing for 18 years, and I don’t know of these ratings. And I’m embarrassed to say I don’t. But even if I did know that the ratings exist—I mean, if it was in front of me, I’d probably find some interest about it, but I still have my favorite cardiologists that I don’t have a problem with and [who have] great bedside manner. And I’m just going to stick with them, you know?” (MMD65; Atlanta, GA).

Instead, PCPs reiterated their trust in their professional network as a principal means of ensuring quality of care for their patients. PCPs also thought of hospital quality measures as being ripe for manipulation and gaming and questioned whether the measures properly accounted for differences in patient severity (Table 3).

#### Perceptions of Factors Driving Disparities in High-Quality Hospital Use

PCPs were unaware of Black-White disparities in high-quality hospital use for elective CABG but agreed that disparities were likely true and widespread. “I do believe these stats… Even if you were to put everything in equal terms, same patients with equal insurance, even sent to the same hospital… the disparity is not just with CABG and cardiac issues. If you dig [into] statistics or if you search in the medical literature, it’s across the board” (MMD54; New York, NY).

However, PCPs saw disparities in high-quality hospital use as mainly driven by patient SDOH (e.g., ability to pay, transportation), patient preferences for racial concordance, and the geographic availability of high-quality hospitals (Table 3).

Finally, PCPs underlined that the national data was unlikely to depict disparities in their health care markets, and emphasized their belief that referral differences were unlikely to reflect provider bias or occur in their own practice. As one PCP stated, “I can speak for our practice. We don’t distinguish on the basis of color. Black, white, yellow, red, purple, it doesn’t—I mean, I really don’t think so. And my referrals are based on the acuteness of the situation and the severity of the situation” (MMD22; New York, NY).

## DISCUSSION

This study examined factors influencing PCPs’ referral decisions to specialty care for CHD treatment and how these factors inform pathways to disparities in high-quality hospital use. Different from prior work, which examines either the referral process itself or specific referral disparities, our focus group discussions began with a general exploration of referral processes and arrived at possible reasons for disparities within this broader context. The emerging themes highlight the complexity of PCPs’ referral decisions and how this complexity may contribute to observed disparities. Main themes are discussed below.

First, our focus group discussions show that, when making referrals, PCPs are faced with an array of competing factors. These factors include patients’ clinical circumstances, access to professional networks, judgements of referral provider quality, and strategies to overcome patient barriers and honor their preferences. Moreover, complex referral decisions are made within the limited time frame of busy practices, and often leave PCPs striving to find the best available vs. absolute best solution for their patients’ needs.

Perhaps the most important study finding is that PCPs continue to rely deeply on their long-standing professional networks when making referrals, even as health system–based networks are increasingly available. While some PCPs do benefit from health system affiliations, the overarching theme of the discussions was that informal, peer-to-peer networks based on mutual trust and respect are the primary driver of CHD treatment referral, overriding other considerations such as published quality measures. Given this central role of informal networks, and the fact that participant PCPs (who served large proportions of Black patients) practiced in areas with high referral network segregation, informal networks emerge as a potential key lever to improve disparities. Thus, interventions facilitating new professional relationships between minority-serving physicians and high-quality institutions, and formal health system affiliations (perceived as an asset by the study PCPs) may provide better structural support for these physicians and improve their patients’ access to high-quality care.

Beyond the critical importance of networks, participating PCPs described significant referral barriers pertaining to patient socioeconomic circumstances (e.g., lack of supplemental insurance, transportation) and preferences for specialist providers. PCPs, however, also described various ways to overcome limitations imposed by SDOH and honor patient preferences by leaning into their professional networks, further underscoring the primary role that their informal networks play in referral decisions.

One persistent issue highlighted by our study is that PCPs are mostly unaware or distrustful of published hospital quality ratings. The finding is in line with older studies showing similar unawareness and distrust.^40^ Given the lack of change over decades, a different approach in how quality measures are presented and disseminated may be needed to achieve physician buy-in and promote their use.

Finally, PCPs acknowledged the existence of widespread health care disparities, but also opined that the CABG-related disparities we presented were telling a story based on national averages that were unlikely to apply to their practice area. Further, they ascribed disparities exclusively to patient and health system factors, including SDOH, preferences for care, and the maldistribution of high-quality hospitals in markets. Some of these factors are confirmed by prior research and addressed by policy, whereas others are not. First, the role of SDOH is widely acknowledged, SDOH screening is being implemented nationwide, and many types of interventions are tested to reduce its impact.^41^ Second, while patient preferences for care have received less attention, prior research found that Black patients have high levels of distrust toward medical institutions^42,43^ and prefer institutions perceived to have higher community engagement.^44^ Therefore, as an investment in better health equity, high-quality medical centers could focus on expanding relationships with vulnerable communities. Finally, the potential role of high-quality hospital maldistribution within local markets as a reason for disparities is less rooted in evidence, with studies suggesting that Black patients live closer to high-quality hospitals than White patients, although they receive care at these hospitals less often.^15,16^

Importantly, participant PCPs did not believe disparities in high-quality hospital use were related to provider bias. This finding is aligned with older studies showing that providers, whether they are PCPs, cardiologists, or cardiac surgeons, tend to attribute disparities in quality of care to patient or system factors (insurance, geographic access) rather than provider factors such as bias.^45–47^ Yet, as other research suggests, referral bias is a potential mechanism for disparities in CHD care.^48^

The study findings should be interpreted in light of its limitations. First, the study focused on three metro areas with high segregation of physician referral networks for cardiac care. Other markets may reveal different drivers of referral decisions and disparities. Second, our focus group discussions centered on limited quantitative data, based on one prevalent condition (CHD) and one referral-based procedure (CABG surgery), as well as one population segment, Medicare beneficiaries. Different factors (e.g., insurance, preferences) may play key roles for younger, working-age populations. Third, we zeroed in on the PCP to cardiologist referral, arguably the first and most important step in the CHD treatment cascade. Future work needs to shed light on other critical aspects of this process, including the views of cardiac care specialists.

Despite these limitations, the study offers evidence that referral decisions remain primarily driven by physicians’ informal networks and by physicians’ perception of patient socioeconomic constraints and preferences. As Black and low-income patients have higher risk for worse CHD outcomes, and high-risk patients fare better in high-quality hospitals,^49^ careful consideration of physician referral as a contributor to disparities is necessary.

## Supplementary Information

Below is the link to the electronic supplementary material.

Supplementary file1 (DOCX 879 KB)

## Data Availability

This is a qualitative study of 6 physician focus groups in three US health markets. All the relevant quotes along with focus group market location and participant study identifiers are included in the supplemental material.
