# Supplementary material for: Referrals and Black-White Coronary Heart Disease Treatment Disparities: A Qualitative Study of Primary Care Physician Perspectives
Source: J Gen Intern Med. 2024 Nov 13;40(15):3572–80. doi: 10.1007/s11606-024-09175-x (PMC12612302; doi:10.1007/s11606-024-09175-x)
Supplement: Supplementary file 1 — Supplementary file1 (DOCX 879 KB) [file 11606_2024_9175_MOESM1_ESM.docx]

**Online Supplement**

**Figure S1.** Segregation of physician patient-sharing networks for cardiac care in the study metro areas

1. **New York**

**
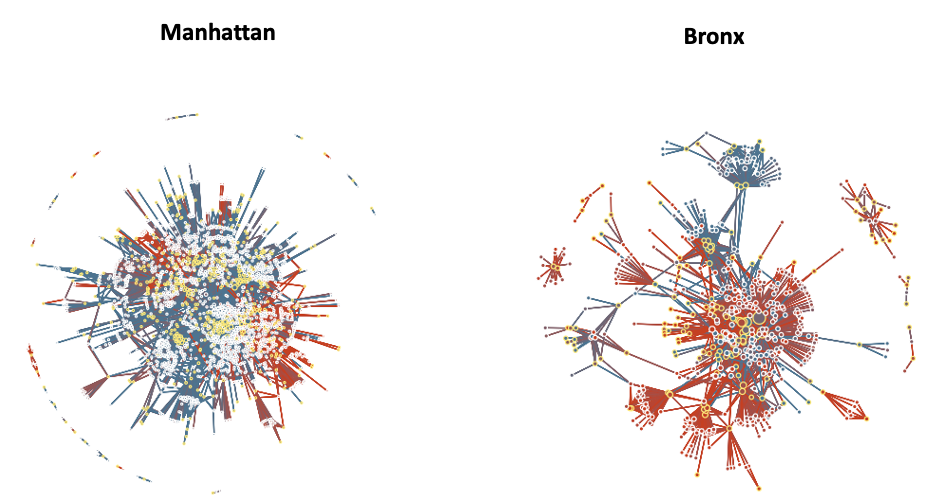
**

1. **Chicago**

**
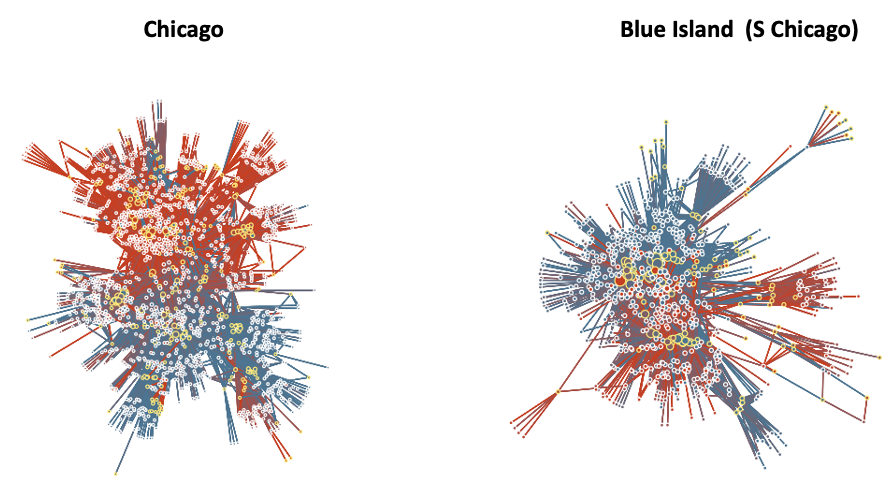
**

1. **Atlanta**

**
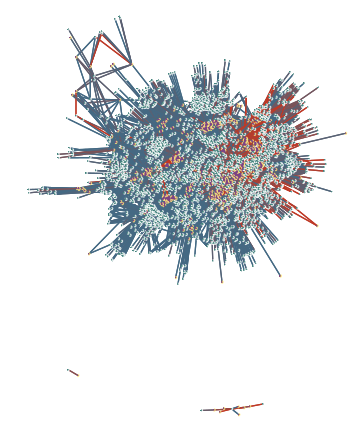
**

**Figures S1 a, b, and c** is a visual representation of the Black-White segregation of referral relationships among PCPs and cardiac care specialists (cardiologists and cardiac surgeons) in the 3 study metro areas and corresponding hospital referral regions (i.e., tertiary markets for cardiac care): (a) New York City, NY (Manhattan and Bronx markets), (b) Chicago, IL (Chicago and Blue Island markets), and (c) Atlanta, GA. We used claims data from 100% fee-for-service Medicare beneficiaries who had heart disease (ischemic heart disease, atrial fibrillation and heart failure) during 2016 -2017 and their treating PCPs and cardiac specialists. We measured relationships between any two physicians as the number of patients they shared, identified via claims submitted by these physicians for the same patients during the 2-year period. The larger the number of shared patients between two physicians, the stronger their network relationship was assessed. A minimum of three shared patients were required to ascertain a relationship between two physicians. We employed quantitative social network analysis methods to depict the relationships between physicians within U.S. hospital referral regions (i.e., tertiary market for cardiac care). We used the R *network* package to visualize each network. In the figures, physicians are represented as circles and the patients they share are represented as ties between circles. Shared Black patients are depicted as red ties, while shared White patients are depicted as blue ties. The clustering of ties by color visually indicates the segregation of Black and White patients for cardiac care within each market.

**Figure S2.** Focus group discussion slide depicting differences in use of high quality hospitals for Black and White patients undergoing elective coronary artery bypass grafting (CABG) surgery and afferent script


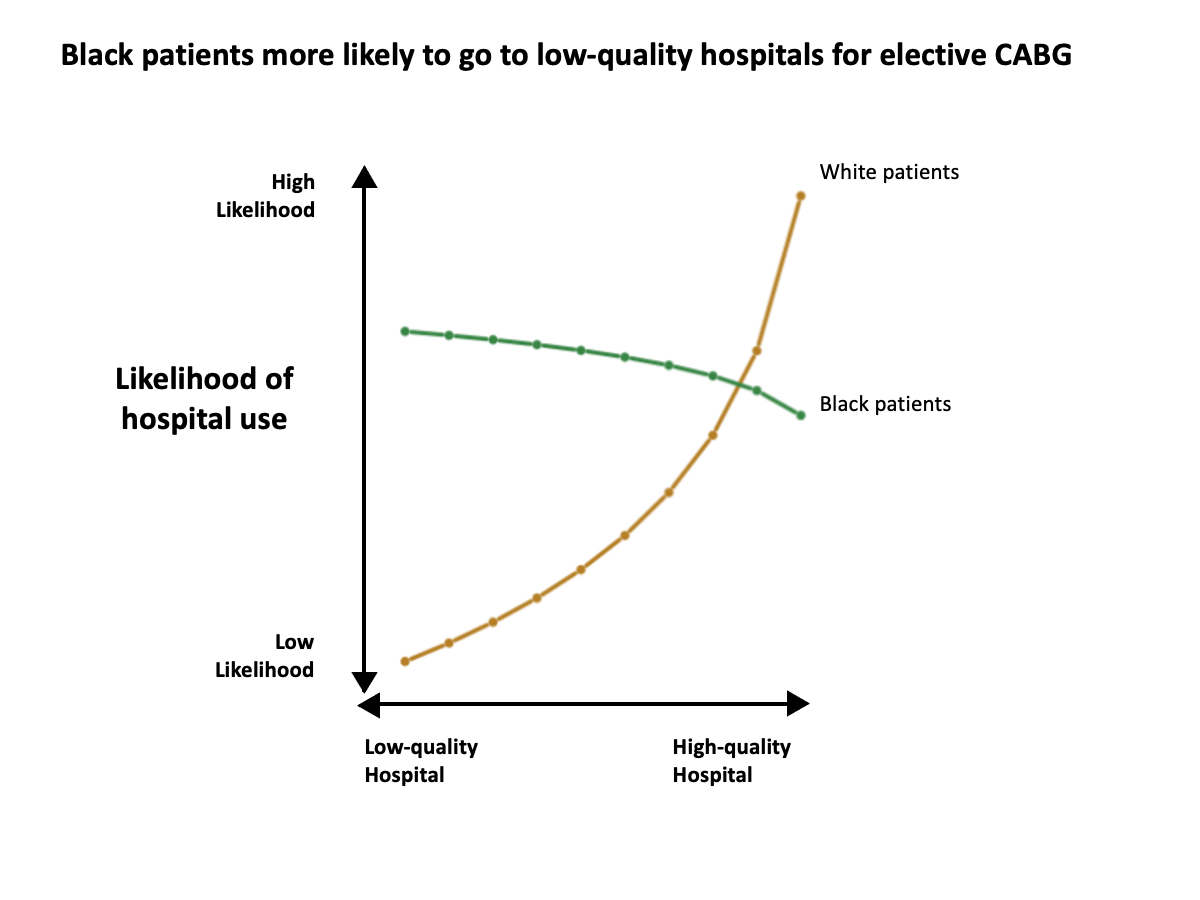


In this slide, the **horizontal axis** is showing hospital quality for patients undergoing elective CABG. Hospital quality was measured using rigorously risk adjusted, hospital level CABG mortality rates. On the horizontal axis hospital quality goes from low on the left to high on the right.

The **vertical axis** is showing the likelihood that a patient is using one of these hospitals, with the top of the graph showing a high likelihood of hospital use, and the bottom showing a low likelihood of hospital use.

The figure represents the universe of Black and White **fee for service Medicare beneficiaries** who underwent **elective CABG** at any CABG performing hospital in the United States. We have focused on these traditional Medicare patients because they all have similar insurance, so insurance should not play a role in the differences we see.

What we see though is that black patients are more or less equally likely to use hospitals at all quality levels (high and low) for CABG procedures, whereas white patients are significantly less likely to use low quality hospitals and more likely to use high quality hospitals. This is after we took into account other factors like how close the hospitals are to where the patients live and hospital CABG volumes.

**Table S1.** Residential and corresponding physician network segregation indexes for study markets

|  | Market-level Segregation Indexes | | | |
| --- | --- | --- | --- | --- |
| HRR | Residential Dissimilarity (D) | Network Dissimilarity (Dn) | Residential Absolute Clustering (ACL) | Network Absolute Clustering (ACLn) |
| Manhattan | 69.6 | 42.6 | 55.3 | 8.1 |
| Bronx | 59.8 | 42.2 | 66.5 | 17.9 |
| Chicago | 76.0 | 65.2 | 63.9 | 29.9 |
| Blue Island | 73.6 | 51.3 | 60.4 | 15.4 |
| Atlanta | 53.9 | 39.5 | 55.5 | 11.2 |

Residential D represents the unevenness of Black and White population distributions across residential zip codes in the HRR. Network Dn represents the unevenness of the Black and White patient distributions across physician network communities (i.e., groups of physicians working together) within the HRR market. Residential ACL is the extent to which majority Black population zip codes adjoin one another (i.e., tend to form enclaves) within the HRR market. Network ACLn is the extent to which physician communities treating predominantly Black patients are connected to other communities treating Black patients vs. communities treating White patients in the market. All indexes range from 0-100, with 0 representing full integration, and 100 representing full segregation. Among all U.S. cardiac care markets with sufficient black population to calculate segregation indexes (169 of 306), the markets selected for this study had network segregation indexes in the highest quintile of the distribution.

**Table S2.** Focus Group Interview Protocol

| **Question** | **Prompts/Probes** |
| --- | --- |
| I’d like to ask first about **urgent referrals**, that is for patients that you think need immediate attention, but that don’t necessarily require an ED visit just based on their history and symptoms. |  |
| What are your main considerations in where you refer a patient for urgent care that day or the next day? | ALLOW MD TO RESPOND AND SAY: I’m going read off some things to you and please tell me how important each one is in where you refer a patient for urgent care   - 1. How quickly they can get care   2. The quality of care and how well you think they will be treated   3. Insurance networks, Medicare Advantage   4. Patient willingness to follow through on referral – what affects that?   5. Maintaining ongoing relationship with patient   6. Relationships with referral networks, other physicians   7. Referral systems where you practice that automatically send a patient to the next available cardiologist |
| How easy or difficult is it for your patients to see a cardiologist for urgent care? Can this normally be done without sending them to an ED or do you have to send them to the ED for this? What determines where you send them? How important is each one of these? | 1. Patient resources and insurance status 2. Access and geographic vs. where the patient lives 3. How easy or hard is usually is to get a quick appointment 4. Patient comfort, willingness to see other physicians, family support 5. Where patients want to go for procedures |
| When you make a referral for urgent care outside the ED do you or does someone on your staff contact the referral doctor directly to make an immediate appointment or do you provide the referral information to the patient or how is this handled? |  |
| Do you have particular cardiologists to whom you regularly make urgent referrals? Why did you choose them? ASK: How important is each one of these things in who you refer to? | 1. Able/willing to see my patients – why? 2. Patient comfort with them, whether they like them – why? 3. Quality of care they provide – how do you monitor that? 4. Where they do procedures – what is important about that? 5. Whether they send the patient back to you or take over care for things you have been treating patients for? |
| Now I’d like to ask about **routine referrals** of patients with coronary heart disease symptoms who can need to be seen by a cardiologist but who can wait a month or so before being seen. |  |
| What are your goals for a referral for routine care? | 1. How quickly they can get care 2. The quality of care and how well you think they will be treated 3. Insurance networks, Medicare Advantage 4. Patient willingness to follow through on referral – what affects that? 5. Maintaining ongoing relationship with patient 6. Relationships with referral networks, other physicians 7. Referral systems where you practice that automatically send a patient to the next available cardiologist |
| How easy or difficult is it for your patients to make an appointment and follow through to see a cardiologist for routine care? What affects whether they do this? | 1. Patient resources and insurance status 2. Access and geographic – what do you mean by this? 3. Patient comfort, willingness to see other physicians, family support |
| When you make a referral for routine care do you have someone on your staff contact the referral doctor and make an appointment or do you provide the referral information to the patient or how is this handled? |  |
| Do you have doctors to whom you regularly make routine referrals? How did you choose them? | - 1. Able/willing to see my patients – why?   2. Patient comfort with them, whether they like them – why?   3. Quality of care they provide – how do you monitor that?   4. Where they do procedures – what is important about that?   5. Whether they send the patient back to you or take over care for things you have been treating patients for |
| Now I’d like to ask about coronary heart disease referrals in general including urgent and routine referrals. |  |
| When you are thinking about referrals for coronary heart disease how important is each of these things about the hospital where the referring doctor does procedures when you decide which cardiologist to suggest for a referral? | - 1. Patient ease of access   2. Patient insurance status   3. Geographic proximity for patients   4. Your professional affiliations   5. Quality of care at that facility |
| Do you have a preferred specialist group where you like to send CHD patients? Why do you prefer them? |  |
| Do you have a preferred hospital or hospitals where you like to have CHD patients go for procedures? Why do you prefer it/them? |  |
| How do you take into account your patients’ preferences when you make a referral? How do you know what they might prefer? Is this something you ask patients about? | IF YES: What do you ask? Please give me an example of this in a cardiologist referral you’ve made? |
| What does “quality of care” mean to you for coronary heart disease patients? What are the indicators of high or low quality care for this condition? What would tell you this case turned out well or turned out badly? |  |
| How do you assess quality of care patients receive from referral physicians and facilities? What sources of information do you have? | 1. Word of mouth, informal reports from staff, other doctors 2. Review of summaries and records of procedures 3. Reports from patients who come back to you, how they are doing 4. Formal ratings |
| What about formal ratings of hospitals for elective CABG procedures such as mortality rates provided by state agencies or Medicare for mortality, complications, readmissions, etc.? Do you ever look at these? | - 1. Do you know where to find them?   2. Do you think they have useful information for making referrals? Why or why not?   3. Is there ever a situation where you might look for formal ratings? When would that be? |
| You’ve described how you do coronary heart disease care and make referrals. How similar or different do you think you are to other primary care doctors in your area? What about older or younger doctors? What about doctors who practice in other parts of the city/area? What about doctors who see different patients in terms of race, ethnicity, income or education? |  |
| We’re very interested in how the way cardiac care referrals are made links to outcomes for patients. What else can you tell me about this that I may not have asked about? |  |

**Table S3.** COREQ Checklist

| **No** | **Item** | **Guide questions/description** | **Response** | **Line Number** |
| --- | --- | --- | --- | --- |
| **Domain 1: Research team and reflexivity** | | | | |
| Personal Characteristics | | | | |
| 1 | Interviewer/facilitator | Which author/s conducted the interview or focus group? | SB and IP | 111-113 |
| 2 | Credentials | What were the researcher's credentials? *E.g. PhD, MD* | PhD and MD, respectively | Cover sheet |
| 3 | Occupation | What was their occupation at the time of the study? | SB, female, expert in qualitative methods and focus group facilitation; IP, female, primary care physician and expert in health services research | 111-113 |
| 4 | Gender | Was the researcher male or female? | Both female | 111-113 |
| 5 | Experience and training | What experience or training did the researcher have? | SB, female, expert in qualitative methods and focus group facilitation; IP, female primary care physician and expert in health services research | 111-113 |
| Relationship with participants | | | | |
| 6 | Relationship established | Was a relationship established prior to study commencement? | Yes; information about the project included in recruitment outreach message | 101-104 |
| 7 | Participant knowledge of the interviewer | What did the participants know about the researcher? e*.g. personal goals, reasons for doing the research* | Yes; information about the project included in recruitment outreach message | 101-104 |
| 8 | Interviewer characteristics | What characteristics were reported about the interviewer/facilitator? e.g. *Bias, assumptions, reasons and interests in the research topic* | Reasons and interests in the research topic | 101-104 |
| **Domain 2: study design** | | | | |
| Theoretical framework | | | | |
| 9 | Methodological orientation and Theory | What methodological orientation was stated to underpin the study? *e.g. grounded theory, discourse analysis, ethnography, phenomenology, content analysis* | Thematic analysis | 125 |
| Participant selection | | | | |
| 10 | Sampling | How were participants selected? *e.g. purposive, convenience, consecutive, snowball* | Purposive sampling | 88-89 |
| 11 | Method of approach | How were participants approached? e*.g. face-to-face, telephone, mail, email* | Email outreach | 101-103 |
| 12 | Sample size | How many participants were in the study? | 72 met criteria, 50 were recruited, 45 participated | 101-104 |
| 13 | Non-participation | How many people refused to participate or dropped out? Reasons? | 5 did not show up for the focus group | 101-104 |
| Setting | | | | |
| 14 | Setting of data collection | Where was the data collected? e*.g. home, clinic, workplace* | Online focus group | 108-109 |
| 15 | Presence of non-participants | Was anyone else present besides the participants and researchers? | No | N/A |
| 16 | Description of sample | What are the important characteristics of the sample? *e.g. demographic data, date* | The characteristics of participating PCPs are described in Table 1. Most participants were male (62.2%), White (57.8%), and practiced for at least 23 years. Participant specialty was either internal medicine (60%) or family medicine (40%) | Lines 140-142; |
|  |  |  |  | Table 1 |
| Data collection | | | | |
| 17 | Interview guide | Were questions, prompts, guides provided by the authors? Was it pilot tested? | Yes, focus group guide included as appendix. Three pilot test interviews were conducted to test the focus group guide | 106-107 |
| 18 | Repeat interviews | Were repeat interviews carried out? If yes, how many? | No repeat interviews | N/A |
| 19 | Audio/visual recording | Did the research use audio or visual recording to collect the data? | Yes, audio recording | 108-109 |
| 20 | Field notes | Were field notes made during and/or after the interview or focus group? | No | N/A |
| 21 | Duration | What was the duration of the interviews or focus group? | 90 minutes | 108-109 |
| 22 | Data saturation | Was data saturation discussed? | No | N/A |
| 23 | Transcripts returned | Were transcripts returned to participants for comment and/or correction? | No | N/A |
| **Domain 3: analysis and findings** | | | | |
| Data analysis | | | | |
| 24 | Number of data coders | How many data coders coded the data? | 2 coders (IP and NQ) | 126-128 |
| 25 | Description of the coding tree | Did authors provide a description of the coding tree? | No | N/A |
| 26 | Derivation of themes | Were themes identified in advance or derived from the data? | Derived from the data | 125-126 |
| 27 | Software | What software, if applicable, was used to manage the data? | Dedoose | 134-135 |
| 28 | Participant checking | Did participants provide feedback on the findings? | No | N/A |
| Reporting | | | | |
| 29 | Quotations presented | Were participant quotations presented to illustrate the themes / findings? Was each quotation identified? e*.g. participant number* | Yes, included throughout and identifier explained in method section | 148 |

**Table S4**: Participant PCP responses regarding factors driving referrals to cardiology services

| **Themes and Subthemes** | **Quote** |
| --- | --- |
| **Specialist vs. hospital choice** | *“It’s typically the cardiologist that knows somebody at that location that will make the phone calls. But if I have a patient that says, “Where is the best place to have a coronary artery bypass graft done?” I’ll say, “I’m sending you to who is the best, who is very good.” I mean, when you say the best hospital, what is it about the hospital? It’s the physician—it’s the physician that really makes the difference there. It’s not just the prestige of the hospital, it’s the physician who’s doing it. So, if there’s somebody at our—and I’m at three different hospitals—if there’s somebody at our hospital that’s equally capable of doing it, I will tell the patient that you’ll get the same standard of care as you would somewhere else. But if it’s something super-specialized, then the cardiologists will make the call to someone in particular.” – M-MD13 (Chicago,* *IL)* |
| **The importance of professional networks** |  |
| PCPs deeply rely on informal professional networks when making referral decisions | “Two cardiologists that I work with […] see probably 90 percent of my patients and I think they respect my judgment and when I ask them to see someone in a pinch, they’ll do so within 24 hours. I think it depends a lot on the relationship you have with the cardiologist—if they know you and have a good relationship with you, then they’re not going to turn your referral away.” - MMD54 (New York, NY) |
| Strong professional relationships are based on trust and mutual respect | “If the patients are happy with the doctor, they have a good bedside manner, that makes me happy […]. Fortunately, I have the same group of doctors for a long time. Once in a blue moon, a new doctor pops in, mostly because the patient has been seeing this doctor and they’re satisfied with the care, so I may refer some patients […].” – FMD55 (New York, NY)  [I go by] “What I see in the notes, [or what] I get to speak to them on the phone […]—you can often tell a lot by just a simple phone conversation, I think.” -MMD53 (New York, NY) |
| Formal affiliations with health systems are desirable, as informal referral networks are becoming increasingly unstable | “I’ve been in this area for over 20 years and have some specific doctors that I refer to […] And it’s been up and down over the years. In fact, maybe a year or two ago, it got to the point where I really didn’t have a go-to person for a while that I could [refer to] in a timely fashion.” – M-MD38 (Atlanta, GA)  [If the patient needs to go elsewhere] “eventually it happens, but it’s not as simple as […] when I do it with my hospital, where I pick up the phone and just make the reservation. You have to [sometimes] present the case to […] the senior resident and then [they] will transmit information to whatever cardiologist on call” – M-MD14 (Chicago, IL) |
| **Valued referral provider characteristics** |  |
| Availability and timeliness of cardiologists | “I find that I have difficulty. And it's become a problem in recent years, in the last couple of years. Because before, I used to be able to call one of my favorite cardiologists and they would be able to accommodate the patient. But lately, it seems...I luck out occasionally […] but for the most part, it's going to be a wait.” – FMD67 (Atlanta, GA)  “I have that problem with the availability of appointments. [If] I call, we can get them in within the next couple days, but oftentimes, it's with a mid-level and not the MD.” – FMD67(Atlanta, GA) |
| Good communication with the cardiologists | “The first couple patients you send, you ask questions when they come back. You look at the [consult] notes and you could […] call and discuss the patient with [the cardiologist]. I think that's good. I've had the cardiology group that I mainly [use] send back even the scan of the blocked artery that I can look at on my phone. So, […] if somebody communicates well with you and [takes] care of the patient […] as soon as possible, […] that tells me that I'm dealing with the correct group.” – FMD66 (Atlanta, GA) |
| ***Consideration of patient circumstances*** |  |
| Geographic proximity and socioeconomic constraints | “If it’s something not as urgent and I don’t think they need to be seen right away, I may defer to what the patient wants in the sense that sometimes they’ll want to see somebody close to their house, so we’ll try to find them somebody in their area.” – MMD53 (New York, NY)  “You need to follow up with the specialist after a [cardiac invasive] procedure, whether it’s a cardiac surgeon, or a cardiologist. [Some patients] will not follow-up. […] They struggle financially, they cannot get there, they don’t have supportive family. So, when you look at the whole picture, it’s still better if they are closer to home because they have better chances for follow-up[…]. And it’s also a trend [to] refer to the places that are closer to you. So, we’re kind of stuck with it. I’ve never seen […] that my colleagues are trying to refer to [hospitals farther away] unless it’s a very specific procedure.” – F-MD15 (Chicago, IL) |
| Insurance limitations | “You know, unfortunately in this day and age, patients’ insurance comes into play. That’s the main thing. What kind of insurance a patient has and what kind of insurance is accepted on the other end. And that will decide which group I’m sending patients to.” - M-MD18 (Chicago, IL) |
| Willingness to lean into informal professional networks | “If it’s somebody who does not have insurance and can’t afford to pay a lot, again I’ll lean on those relationships. […] I know some cardiologists that will charge lower out-of-pocket costs to people who can’t afford it, so I’ll utilize those services in those cases.” – FMD35 (Atlanta, GA)  “If the patient has [poor insurance], then it's going to be a tough call. Patients may want to go someplace, but that place may not accept this insurance. So, you have to really look into and probably call that system and see if they would accept the insurance. Then, probably you have to do some homework and see where you can refer the patient. [That] is a big burden.” – FMD42 (Chicago, IL) |
| Honoring patient preferences or desire to avoid particular providers | “A lot of [patients] care about which system they end up in because they may […] have had a bad experience with [Hospital #1]. And then, they're like, okay, don't send me to that system.” – MMD38 (Atlanta, GA)  “Some of my patients do have those requests and [and] the cardiologists that have in my Rolodex are in different hospital systems, too. So, it does work out that can [refer them to] someone [at] their preferred facility.” – FMD34 (Atlanta, GA) |
| Overriding patient preferences given concerns about quality of care | “There are some hospitals [that] handle cardiac care better than others. So, depending upon [the patient severity], that makes an impact on where I might direct the patient over where they feel they may be the most comfortable.” – F-MD35 (Atlanta, GA)  “A lot of my patients are Latino, [and] I have to sacrifice [medical care] in Spanish in favor of the outcome. I prefer the outcome versus the comfort of the patient, in that sense.” – M-MD14 (Chicago, IL) |

**Table S5.** Participant PCP responses regarding referral provider quality and disparities in the quality of hospitals used for CHD treatment

| **Themes and subthemes** | **Quote** |
| --- | --- |
| **Opinions on hospital quality measures** |  |
| PCPs lack awareness of published data while professional relationships weight heavily in hospital quality assessments | “I’ve been practicing for 18 years, and I don't really know of these ratings. And I'm embarrassed to say I don't. But even if I did know that the ratings exist—I mean, if it was in front of me, I'd probably find some interest about it, but I still have my favorite cardiologists that I don't have a problem with and [who have] great bedside manner. And I'm just going to stick with them, you know? Honestly similar to what some of the others said, I don't really care, if I already feel comfortable […] using the cardiologist who I have had a relationship [with] over the years.” – MMD65 (Atlanta, GA) |
| Lack of trust in published quality data | “So [there is] all of the data that you can get, but it’s hard to judge. I mean, even looking for a doctor for myself, it’s very hard to judge [...] the information out there, what you see on a website, what’s reported, if there are ways to game systems, it’s hard to know, I think, in many cases. So, you sort of do the best you can.” – MMD53 (New York, NY)    “This data can be manipulated. And so, the health system that's providing the data can manipulate the data the way that they want” – MMD69 (Atlanta, GA) |
| Published quality assessments ignore important confounders | “You know, those formal ratings, even though you might think they’re supposedly objective, they’re based on outcomes, but if [those hospitals] are taking care of much more complicated patients[ …] they’re going to have a little bit worse outcomes, perhaps, because of the patient population they’re taking care of. So, ratings that may seem objective aren’t necessarily all objective.” – FMD11 (Chicago, IL) |
| **Opinions on factors driving disparities in *high-quality hospital use*** |  |
| Patient factors | “Maybe they've had a personal experience with a family member. [Maybe] they've actually been to that hospital and if they're going to have an elective procedure like CABG, they want to feel comfortable.” – MMD45 (Chicago, IL)  “I also think access […] plays a hug role in this, as well. If patients are in a particular community of hospitals, and they don’t necessarily know their rating scores. They're just going to go where [access] is offered that’s near versus driving 30 miles across town to go to a higher quality hospital. Again, depending on socioeconomic status and ability to pay” - FMD64 (Atlanta, GA)  ‘The low-quality hospitals are probably more likely in the areas where more Black people live, [and patients are likely to go] someplace that is close to them, [where...] employees […] are more likely Black as well. So, all those things come into play.” - MMD63 (Atlanta, GA) |
| System factors |  |
| Race-based referral differences are unlikely | “I can speak for our practice. We don't distinguish on the basis of color. Black, white, yellow, red, purple, it doesn't—I mean, I really don't think so. And my referrals are based on the acuteness of the situation and the severity of the situation” – M-MD22 (New York, NY) |
